# Design and Characterization of Liposomal-Based Carriers for the Encapsulation of *Rosa canina* Fruit Extract: *In Vitro* Gastrointestinal Release Behavior

**DOI:** 10.3390/plants13182608

**Published:** 2024-09-18

**Authors:** Aleksandra A. Jovanović, Bojana Balanč, Predrag M. Petrović, Mina Volić, Darko Micić, Jelena Živković, Katarina P. Šavikin

**Affiliations:** 1 Institute for the Application of Nuclear Energy INEP, University of Belgrade, Banatska 31b, 11080 Belgrade, Serbia; 2 Innovation Centre of the Faculty of Technology and Metallurgy, University of Belgrade, Karnegijeva 4, 11000 Belgrade, Serbia; bisailovic@tmf.bg.ac.rs (B.B.); ppetrovic@tmf.bg.ac.rs (P.M.P.); mvolic@tmf.bg.ac.rs (M.V.); 3 Institute of General and Physical Chemistry, Studentski trg 12/V, 11158 Belgrade, Serbia; dmicic@iofh.bg.ac.rs; 4 Institute for Medicinal Plants Research “Dr Josif Pančić”, Tadeuša Košćuška 1, 11000 Belgrade, Serbia; jzivkovic@mocbilja.rs (J.Ž.); ksavikin@mocbilja.rs (K.P.Š.)

**Keywords:** functional foods, liposomes, polyphenol release, rosehips, thermal properties

## Abstract

The increasing demand for natural compounds as an alternative to synthetic antioxidants and conservans has led to the utilization of secondary plant metabolites in the food industry, as these bioactive compounds possess great antioxidative and antimicrobial properties without side effects on human health. Despite this, the sensitivity of plant-derived compounds is a restrictive factor in terms of their full potential. The current research aimed to characterize rosehip-fruit-extract-loaded liposomes (non-treated and UV-irradiated) in terms of their density, surface tension, viscosity, chemical composition (FTIR and HPLC analyses), and thermal behavior. In the storage stability study, the vesicle size, polydispersity index (PDI), zeta potential, conductivity, and mobility of the liposomes were monitored. FTIR analysis confirmed that the plant compounds were successfully loaded within the carrier, while no chemical reaction between the rosehip fruit extract and phospholipids was detected. The results of the HPLC analysis evidence the high potential for liposomal encapsulation to protect sensitive bioactives in the rosehip fruit extract from the degrading effect of UV irradiation. The size of the rosehip-fruit-extract-encapsulated liposomes increased on the seventh day of storage from 250 nm to 300 nm, while the zeta potential values were between −21 mV and −30 mV in the same period and further stabilized over 60 days of monitoring. *In Vitro* release studies in water and simulated gastrointestinal fluids showed that the presence of enzymes and bile salts (in intestinal fluid) enhanced the rosehip–polyphenol permeability from liposomes (70.3% after 6 h) compared with their release in water after 24 h and in gastric fluid after 4 h (38.9% and 41.4%, respectively). The obtained results indicate that the proliposome method was an effective method for rosehip fruit extract liposomal encapsulation and for the delivery of these plant-derived bioactives in foods.

## 1. Introduction

Over the last decade, consumer awareness about foods’ contribution to their health has increased, so the requirements in food production have changed considerably. Apart from their nutritional properties, foods are intended to prevent diseases and improve overall well-being. Innovations in the food industry mainly refer to new approaches in food processing and the introduction of novel foods. Among all these innovations, the development of functional foods of plant origin is recognized as one of the most interesting [1,2].

Plants represent a rich source of bioactive compounds that provide health benefits and possess strong antioxidative properties. Considering that synthetic components and/or drugs have demonstrated significant side effects, there has been a demand for alternatives found in bioactive compounds from plants that have been active in the treatment of many diseases [3]. Among these, polyphenolic compounds have become an emerging field of interest in food and nutrition, as they may be crucial in the regulation of metabolism, chronic diseases, and cell proliferation [4,5]. Namely, free-radical damage seems to be somewhat limited by the action of polyphenols, which are natural antioxidants present in daily food.

Polyphenol-enriched functional foods are the main topic of current research. Wild rosehip, *Rosa canina* L., a plant from the Rosaceae family, is rich in polyphenolic compounds, including flavonoids, anthocyanins, and phenolic acids [6]. Moreover, *R. canina* is a valuable source of vitamins [7], carotenoids, tocopherols, amino acids, minerals, and organic acids [8]. Hence, rosehip has shown great antioxidative, anti-inflammatory, antibacterial, antinociceptive, and anti-cancerogenic properties [9], which indicates its great potential to be employed in the production of functional plant foods. Despite polyphenols having a wide range of beneficial effects on human health, their use is limited due to the lack of long-term stability, poor bioavailability, and sensitivity of these compounds to light, oxidation, and high temperatures [5].

The technologies for encapsulation are promising in protecting sensitive bioactive compounds from adverse environmental conditions, as well as in promoting a controlled and targeted liberation of the encapsulates [10]. Among the many encapsulation techniques, liposomal entrapment is one of the most suitable, as its major advantage is the ability to control the release rate of the encapsulated compounds and to deliver them to the right place at the right time [11]. Moreover, encapsulation in liposomes ensures good protection from stomach digestion and enhances the bioactivity and bioavailability of active substances [12].

Furthermore, UV irradiation using bactericidal ultraviolet lamps is widely applied commercially in the food industry to slow down the aging of meat and different types of cheese and to prevent surface mold growth on bakery products, as well as for air purification during bottling and food processing [13]. UV irradiation possesses germicidal properties at 250–270 nm (UV-C region) that are widely used in the food or pharmaceutical industries. The high energy associated with a short wavelength can kill or disable bacteria, viruses, and protozoa due to changes in cellular nucleic acids, breaking the molecular bonds in DNA within microorganisms, creating new bonds among nucleotides, and producing thymine dimers [14].

The objective of this work was to offer a liposome-based carrier system for rosehip polyphenols with a possible application as an additive in the food industry. *R. canina* has already been encapsulated in liposomes but by using a thin-film method and for dermo-cosmetic applications [15]. The proliposome method of entrapment used in this work has several benefits compared with the thin-film method due to the possibility for scale-up as a significant approach in industrial-level food production, the smaller diameter size of the liposomes, and the non-utilization of chloroform [11].

Characterization of rosehip-fruit-extract-loaded liposomes (non-treated and UV-irradiated) was carried out in terms of their density, surface tension, viscosity, and chemical composition (via Fourier-transform infrared, i.e., FTIR and HPLC analyses). In food production, e.g., during baking, high temperatures may influence the stability of liposomal systems; thus, thermogravimetric analysis (TGA), as a key in the investigation of the thermal behavior of the carriers, was conducted. The last step in food production, which may involve the removal of microbes on the surface of food and/or food package, usually involves UV irradiation, so the characterization of UV-treated samples was also performed. In addition, the vesicle size, polydispersity index (PDI), zeta potential, mobility, and conductivity of the liposomes were monitored in a storage stability study for 60 days. The *In Vitro* polyphenol release behavior in water and simulated gastric and intestinal fluids with corresponding enzymes was examined in the Franz diffusion cell to assess the polyphenol release kinetics, diffusion coefficient, and diffusion resistance.

## 2. Results and Discussion

In the present research, liposomal vesicles with *R. canina* fruit extract were developed and characterized in terms of storage stability, UV stability, density, surface tension, viscosity, chemical composition (FTIR and HPLC analyses), thermal behavior (TGA), and polyphenol release in water and simulated gastrointestinal conditions.

### 2.1. Storage Stability of Liposomes

**Figure 1:**
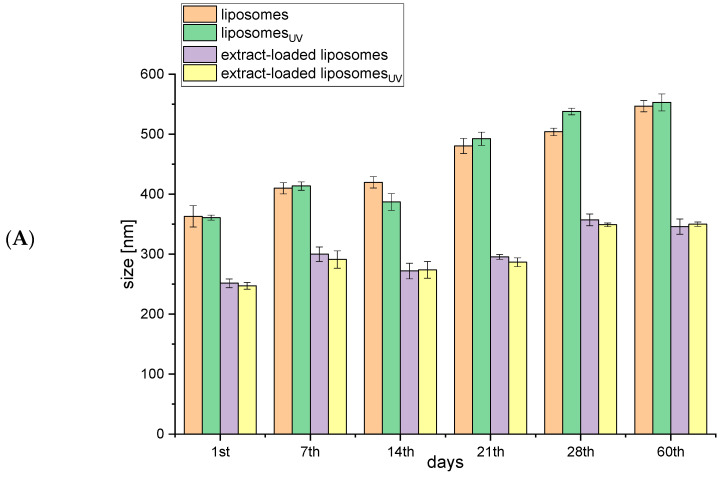

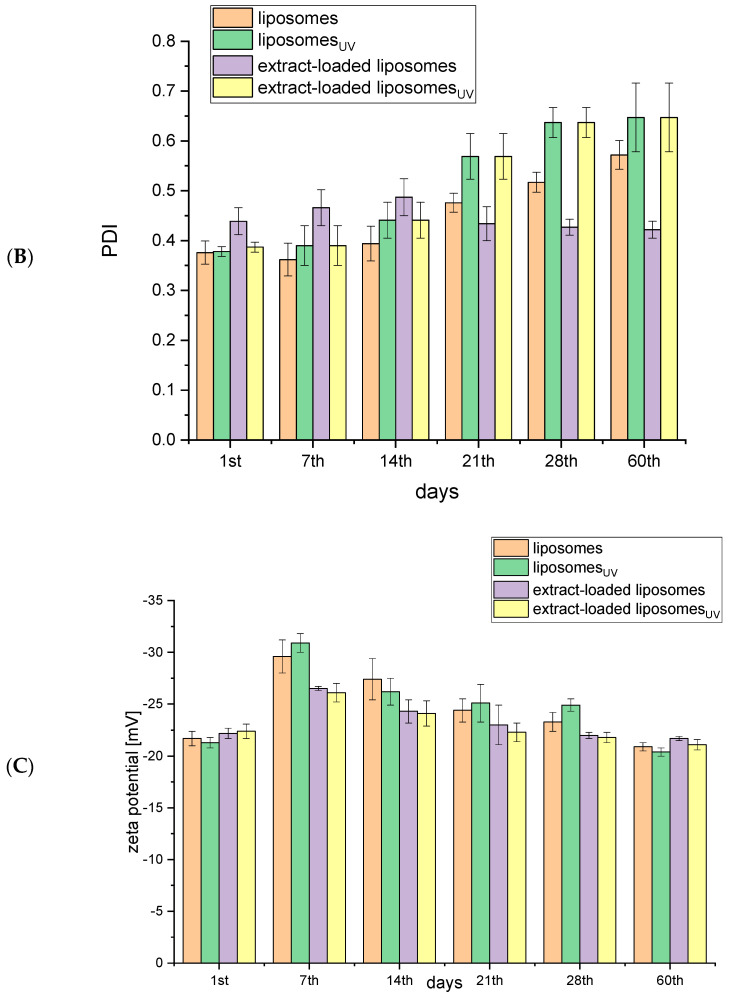

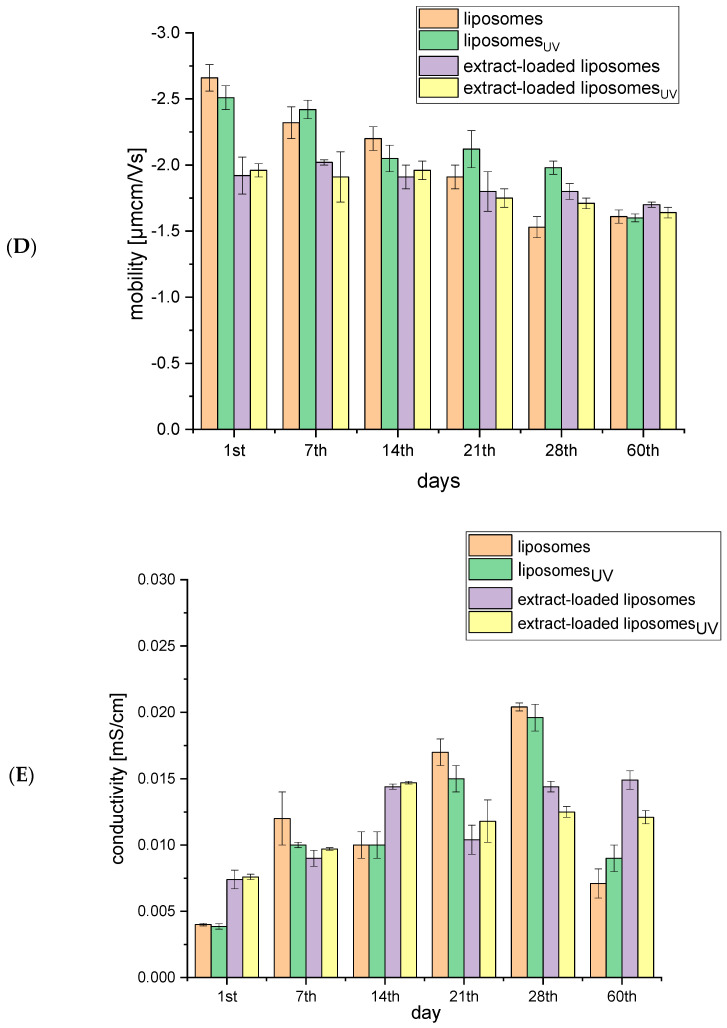
Particle size (**A**), polydispersity index (PDI) (**B**), zeta potential (**C**), mobility (**D**), and conductivity (**E**) of empty and *Rosa canina*-fruit-extract-loaded liposomes (non-treated and UV-irradiated) over 60 days of storage at 4 °C (mean value ± SD); liposomes_UV_, UV-irradiated empty liposomes; extract-loaded liposomes_UV_, UV-irradiated liposomes with extract.

As liposomes are not thermodynamically stable systems, their application as nanoscale carriers requires the monitoring of their stability, which is reliably characterized by photon correlation spectroscopy, which includes particle size, size distribution, and zeta potential measurements [16]. To estimate the storage stability of the obtained liposomes (non-treated and UV irradiated empty and rosehip-fruit-extract-loaded liposomes), particle size, PDI, zeta potential, mobility, and conductivity were measured over the 60 days of storage at 4 °C, and the results are presented in Figure 1.

The size of lipid vesicles, as a parameter related to their structure and composition, can increase before the appearance of macroscopic changes in liposomal suspensions [16]. The dimension changes indicate the instability of liposomal systems; thus, the size of empty and *R. canina*-fruit-extract-loaded liposomes (non-treated and UV-irradiated) was monitored for 60 days. An increase in the diameter of empty liposomes (non-treated and UV-irradiated), from 360 nm to 410 nm, was detected by the 7th day of storage in refrigeration, and the particle size continued to increase continuously up to the 60th day, to 550 nm (Figure 1A). The size of rosehip-fruit-extract-loaded liposomes (non-treated and UV-irradiated) increased by the 7th day, from 250 nm to 300 nm, and it did not change between the 7th and 21st day of storage (Figure 1A). The second increase in the diameter was detected on the 28th day, when it measured 350 nm, and that value remained the same on the 60th day. Since the same trend was observed for both non-treated and UV-irradiated liposomes, it can be concluded that irradiation did not cause the additional destabilization of the liposomal bilayer or any disturbance in the stability during storage. A study that dealt with the supercritical-assisted encapsulation process for olive pomace extract into liposomes demonstrated that the size of the obtained liposomes remained constant for more than 120 days [17]. On the other hand, liposomes produced by the conventional method were not stable during the prolonged storage period due to aggregation and phase separation. Dag and Oztop also showed an increase in the mean vesicle size of liposomes obtained using ultrasound waves on the seventh day of storage [18]. This phenomenon can be clarified by the fact that small particles formed by sonication are mostly metastable and can grow over a while to decrease the high curvature energy associated with the large bending of the lipid bilayer. Additionally, Taylor et al. suggested that ultrasound energy distribution throughout the liposomal suspension was non-homogenous during sonication, which consequently resulted in less uniform or often multi-modal vesicle size distributions [19]. The incorporation of sterols, including cholesterol, β-sitosterol, ergosterol, lanosterol, etc., within the liposomal membrane can improve the stability of liposomes by increasing their zeta potential, *i.e.*, the electrostatic repulsion between particles, and, consequently, prevent fusion and aggregation of the liposomal vesicles [20]. Moreover, the release rate and liposome stability can be adjusted or controlled by the addition of different surfactants [21]. Nonionic surfactants are mostly used for this purpose since they have low toxicity and surface activity [22,23].

Since liposomes should be as homogeneous as possible to avoid undesirable coalescence, the size distribution, as an important parameter characterizing the stability of colloidal dispersion [16], was determined over 60 days of storage. The PDI values are given in Figure 1B. PDI values of empty liposomes (0.370 for non-treated and 0.390 for UV-irradiated), representing a narrow distribution, slightly increased (0.400 for non-treated and 0.440 for UV-irradiated) after the storage period of 14 days and significantly and continuously increased until the 21st day and again until 60th day (0.480–0.570 for non-treated and 0.570–0.650 for UV-irradiated). On the other hand, the PDI of the rosehip-fruit-extract-loaded liposomes did not change during the 60 days of storage (Figure 1B), as in the case of phospholipid/sterol liposomes with wild thyme extract [24]. The obtained difference between the empty and rosehip-fruit-extract-loaded liposome PDI values agrees with the literature data, where Gibis et al. reported that empty liposomes possessed higher PDI values (indicating a wider particle size distribution) after storage due to the oxidative degradation of unsaturated fatty acids [25].

The data for the particle size and PDI values obtained during the stability study agree with the results of the surface tension analysis discussed in the next section (Section 2.2). Namely, when flavonoids presented in the rosehip fruit extract are adsorbed at the lipid–water interface, it is difficult to remove them from the surface, which makes the emulsion stable against particle variations and migration [26]. Therefore, rosehip-fruit-extract-loaded liposomes exerted lower surface tension and better stability, and the examined parameters (particle size and size distribution) did not change as much as for empty liposomes.

The zeta potential is a fundamental parameter for the evaluation of repulsion forces between liposomal particles and, therefore, of the stability of the liposomal system. According to the literature, when the zeta potential of a liposomal suspension is approximately −30 mV or +30 mV, it indicates its satisfactory stability during prolonged storage [16]. In the present study, the zeta potential varied in all the liposomal suspensions during the 60-day stability study (Figure 1C). In all the samples, the zeta potential (absolute value) statistically significantly increased on the seventh day from −21 to −30 mV for empty liposomes and from −22 to −26 mV for rosehip-fruit-extract-loaded liposomes. Guldiken et al. demonstrated that samples with a higher absolute value of the zeta potential do not aggregate due to electric repulsion [27]. After the 14th day, the zeta potential started to decrease continuously, and on the 60th day, it attained approximately the same values as on the 1st day, namely −20 mV for the empty liposomes and −21 mV for the rosehip-fruit-extract-loaded liposomes. The literature data also demonstrated that zeta potential values decreased on the 14th day for green-tea-extract-loaded liposomes [18]. Since the fact that the zeta potential value is used as a measure of the liposomal system stability, the obtained changes in the surface charge may indicate the appearance of some modifications within the liposomes. However, in the case of liposomes with green tea extract, the decrease in zeta potential was followed by the change in polyphenol content on the 14th day of storage [18], which was not the case with the rosehip-fruit-extract-loaded liposomes, where there was no leakage of polyphenols even after 60 days of storage (the same concentration of polyphenols in the supernatant as on the 1st day).

According to the results from Figure 1D, it can be observed that the mobility (absolute value) decreased over 60 days, particularly in the case of the empty liposomes (from −2.66 to −1.61 µmcm/Vs for non-treated and from −2.51 to −1.60 µmcm/Vs for UV-irradiated). The decrease in mobility of rosehip-fruit-extract-loaded liposomes was lower, from −1.92 to −1.70 µmcm/Vs for non-treated and from −1.96 to −1.64 µmcm/Vs for UV-irradiated samples. The interactions between liposomes, which depend, among other things, on the quantity of liposomes per milliliter, affect liposome modifications, as well as changes in mobility. Therefore, liposome fusion or fission can result in the redistribution of phospholipids between liposomes and cause variations in mobility [28]. The obtained results of the mobility changes during storage are consistent with the above statement because there were also changes in vesicle size in all the liposomal samples, particularly in empty liposomes (Figure 1A).

Since changes in conductivity indicate whether liposomes have fused (shown by a decrease in conductivity) or are leaking (shown by an increase in conductivity), this parameter was also measured during a 60-day stability study (Figure 1E). In all the liposomes, the conductivity increased after 7 days and varied in the range of 0.01 to 0.02 mS/cm (for empty liposomes) and from 0.009 to 0.015 mS/cm (for rosehip-fruit-extract-loaded liposomes). However, the conductivity had decreased by the 60th day, but only for empty liposomes, which agrees with the vesicle size changes during storage, which were more pronounced in the mentioned sample (Figure 1A). Therefore, the decrease in conductivity seen within a liposome dispersion upon a vesicle size increase can be directly related to the water volume captured by the liposome [29].

### 2.2. Physical Properties of Rosa canina Fruit Extract and Liposomes

The physical properties of the *R. canina* fruit extract and of empty and extract-loaded liposomes (density, surface tension, and viscosity) were investigated before and after UV irradiation; the results are presented in Table 1.

To meet challenges and to develop new and better-performing products in the food, pharmaceutical, and cosmetic industries, knowledge of physical properties, such as density and surface tension, is of fundamental importance, particularly for the standardization and characterization of liposomal products [30]. Namely, the surface tension of liposomes should be determined to find the optimal ratio of phospholipids/active compounds for maximal release and stability. Hence, the density and surface tension of empty and rosehip-fruit-extract-loaded liposomes (before and after UV irradiation) were monitored. UV irradiation did not influence the two above-mentioned variables in the liposomal samples. However, in the case of the *R. canina* fruit ethanol extract, the evaporation of ethanol during UV irradiation caused changes in the density, surface tension, and viscosity values. As can be seen from Table 1, the density of empty liposomes was lower (0.998 ± 0.000 g/mL) in comparison with extract-loaded liposomes (1.010 ± 0.002 g/mL) but higher than that of pure rosehip fruit extract (0.931 ± 0.001 g/mL). Further, empty liposomes possessed a significantly higher value of surface tension (24.1 ± 0.1 mN/m) compared with the *R. canina*-fruit-extract liposomes (18.7 ± 0.3 mN/m). The surface tension of pure rosehip fruit extract (25.4 ± 0.2 mN/m) was statistically significantly higher than that of the liposomal samples. Azarbayjani et al. demonstrated that the surface tension of liposomes is related to the phospholipid content and depends on the characteristics and concentration of encapsulated compounds as well [30]. A study that dealt with liposomal particles with an antifungal compound showed that the addition of high miconazole content caused a decrease in the surface tension of liposomes [31]. The obtained results are in agreement with the literature observation that polyphenols presented in the extracts can slightly decrease the tension at the oil–water interface and, consequently, inhibit lipid oxidation, but polyphenols cannot significantly improve the stability of the system [26]. On the other hand, Luo et al. reported that flavonoids have a role as good stabilizers of emulsions due to their adsorption at the surface [32]. This can be clarified by the partition coefficients of flavonoids somewhat influencing their surface activity and thereby inducing good emulsion stability [26,32]. The obtained results are in good correlation with the data from the stability study discussed in the previous section (Section 2.1.). The viscosity of all the investigated samples varied from 3.04 to 6.75 mPa·s (Table 1). The lowest values of viscosity were determined for the empty liposomes, while the addition of the rosehip fruit extract significantly increased the mentioned variable. UV irradiation did not cause a significant change in the viscosity of the liposomal samples. Demirbay et al. have also shown that UV radiation did not influence the viscosity behavior of the solutions [33].

### 2.3. FTIR Spectra of Rosa canina Fruit Extract and Liposomes

**Figure 2:**
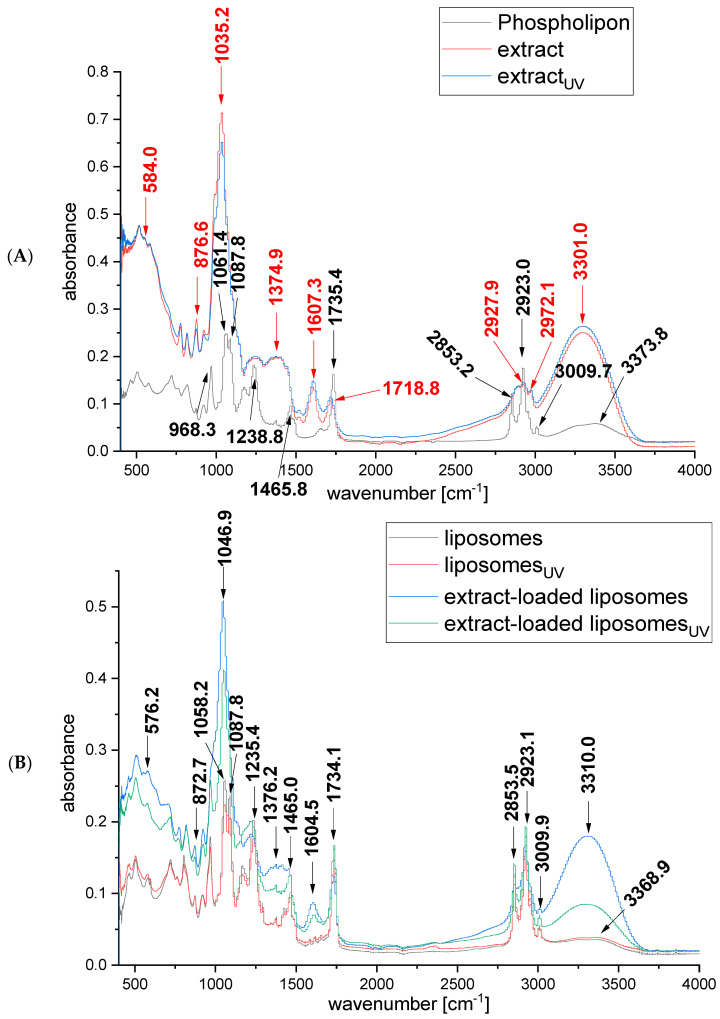
FTIR spectra of (**A**) the initial components, Phospholipon, and lyophilized *Rosa canina* extract, and (**B**) lyophilized empty liposomes and extract-loaded liposomes (before and after UV irradiation); extract_UV_, UV-irradiated extract; liposomes_UV_, UV-irradiated empty liposomes; extract-loaded liposomes_UV_, UV-irradiated liposomes with extract.

FTIR spectroscopy was applied to analyze and investigate the presence of different interactions between the rosehip fruit extract and liposomes. The FTIR spectra of Phospholipon, the non-treated and UV-irradiated rosehip fruit extracts, and the empty and extract-loaded liposomes are presented in Figure 2.

The FTIR spectra of *R. canina*-fruit-extract-loaded liposomes (Figure 2B) mostly resembled the FTIR spectra of empty liposomes (Figure 2A), meaning that most of the peaks corresponding to the extract (Figure 2A) overlaid with the peaks of phospholipids. In the case when the FTIR spectra show mostly peaks originating from the constituent components of the carrier, it can be concluded that the target compounds are successfully loaded within the carrier, i.e., liposomes in this case [34]. The differences between empty and rosehip-fruit-extract-loaded liposomes are visible in the positions of the bands at 3310 cm^−1^, 1604.5 cm^−1^, and 1046.9 cm^−1^ (Figure 2B). The peak at 3310 cm^−1^ could be related to lipids from the rosehip fruit extract and -OH group vibrations as well [35,36]; the peak at 1604.5 cm^−1^ shows the aromaticity behavior of the flavonoids [37]; the peak at 1046.9 cm^−1^ can originate from the ketone group [38]. However, the broad peak at 3300 cm^−1^, which corresponds to free -OH in molecules and -OH groups forming hydrogen bonds in macromolecular associations, has appeared in FTIR spectra in several studies dealing with gold, silver, and palladium nanoparticles containing rosehip extract [36,39,40]. The following peaks were not visible in FTIR spectra of rosehip-fruit-extract-loaded liposomes, but they appeared in the FTIR spectra of pure rosehip fruit extract: the peak at 2972.1 cm^−1^ is specific to the C-H stretching frequency, and the peak at 1718 cm^−1^ may be related to carbonyl group (C=O) stretching vibrations in ketones, aldehydes, and carboxylic acids [36,41]. On the other hand, peaks appearing in both the FTIR spectra of liposomes and FTIR spectra of the rosehip fruit extract at 2925 cm^−1^ may originate from stretching C-H vibrations [36,42], while the relatively weak band at 1375 cm^−1^ corresponds to the umbrella deformation vibrations of the CH_3_ groups of alkyl chains [43], and peaks at 875 cm^−1^ and 580 cm^−1^ are specific for δ C-H in-plane deformation vibrations [38]. The following spectral bands originate only from phospholipids: a broad band at 3368.9 cm^−1^ corresponds to the O-H stretching in water molecules associated with membranes via hydrogen bonds; bands at 3009.5 cm^−1^ and 2853.5 cm^−1^ represent C-H stretching; the band at 2955 cm^−1^ is specific for the antisymmetric stretching vibration in CH_3_ groups; the band at 1735 cm^−1^ represents the stretching vibrations of the ester carbonyl groups; the band centered at 1465 cm^−1^ is assigned to the scissoring vibrations of CH_2_ groups; the band at 1235.4 cm^−1^ is specific for the antisymmetric stretching of PO_2_^−^ groups; and the band at 1087.8 cm^−1^ corresponds to symmetric PO_2_^-^ stretching and partially overlaps with the band at 1058.2 cm^−1^ that represents C-O-P-O-C stretching [36,43]. Furthermore, the absence of new bands on the FTIR spectra of liposomes with rosehip fruit extract compared with the FTIR spectra of unloaded liposomes (Figure 2B) indicates that there is no chemical reaction between the rosehip fruit extract and phospholipids, thus indicating their compatibility. Additionally, there is no difference between the FTIR spectra of non-treated and UV-irradiated extract and liposomes, showing that no new functional groups were created under the influence of irradiation.

### 2.4. Polyphenol Profile of Rosa canina Fruit Extract and Extract-Loaded Liposomes

The quantification of polyphenols in *R. canina* fruit extract and extract-loaded liposomes (before and after UV irradiation) was carried out using the HPLC method. The results are shown in Table 2. The polyphenol compounds quantified in native *R. canina* fruit extract were chlorogenic acid, rutin, and quercetin derivatives, including hyperoside, isoquercetin, and quercetin. According to the literature data and HPLC analyses, biologically active compounds from the extracts of rosehip include, among others, rutin, quercetin, isoquercetin, hyperoside, and chlorogenic acid [44,45,46,47]. However, UV irradiation causes a significant decrease in the content of all polyphenol compounds. Bąkowska et al. reported that UV irradiation had a stronger degradation effect on complexes that possessed flavonoids, such as rutin and quercetin, and phenolic acids, such as chlorogenic acid [48].

As can be seen from Table 2, UV irradiation caused a significant decrease in the content of chlorogenic acid, rutin, and quercetin derivatives (hyperoside, isoquercetin, and quercitrin) in the pure rosehip fruit extract. The most dominant effect was in the case of rutin, where UV irradiation decreased its concentration by 64%, followed by quercetin derivatives (hyperoside, quercitrin, and isoquercetin), where the contents were reduced by 45%. The obtained results, where the reduction in the content of quercetin derivatives was lower than that in rutin, are in agreement with the literature data proving the higher UV protection of quercetin and its derivates in comparison with rutin [49]. The influence of UV irradiation on the chlorogenic acid concentration was significantly lower, and the decrease only amounted to 15%. The resistance of chlorogenic acid in the presence of UV irradiation is not surprising considering that the mentioned acid has shown photoprotection against UV-induced skin damage using *In Vitro* skin models [50]. At the same time, there were no differences in the content of polyphenols between non-treated and UV-irradiated rosehip-fruit-extract-loaded liposomes (Table 2). The fact that the content of chlorogenic acid, rutin, and quercetin derivates (hyperoside, isoquercetin, and quercitrin) was similar in both liposomal samples (non-treated and UV-irradiated) evidences the high potential for liposomal encapsulation to protect sensitive bioactives in the rosehip fruit extract from the degrading effect of UV irradiation.

### 2.5. Thermal Stability of Rosa canina Fruit Extract and Liposomes

**Figure 3:**
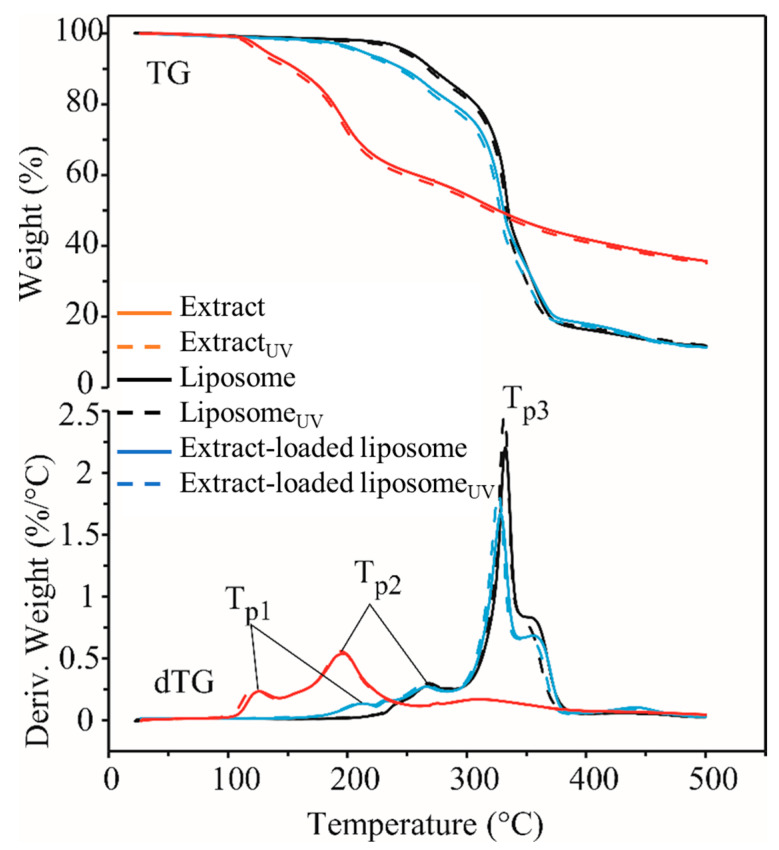
Thermogravimetric (TG) and differential TG (dTG) curves of the *Rosa canina* fruit extract, liposomes with and without loaded extract, and their UV-treated counterparts; extract_UV_, UV-irradiated extract; liposomes_UV_, UV-irradiated empty liposomes; extract-loaded liposomes_UV_, UV-irradiated liposomes with extract.

The TG/dTG curves of all the samples are shown in Figure 3, and the obtained results are listed in Table 3. *R. canina* fruit extract samples proved to be the least thermally stable (decomposition started at about 104–108 °C), while the empty liposome samples were the most thermally stable (decomposition started at about 225–228 °C). Thermal decomposition of the rosehip-fruit-extract-loaded liposome samples started at about 177 °C. UV treatment did not affect the thermal stability of empty and rosehip-fruit-extract-loaded liposomes (*p* > 0.05), while the UV-treated extract was less thermally stable than the non-treated parallel at about 4 °C (*p* < 0.05). The decomposition process was completed by 386 °C for the rosehip fruit extract, while for the liposome samples (empty and extract-loaded), it lasted until 372–378 °C. Weight loss (WL) during the decomposition process was 58–59% for the rosehip fruit extract and 80–82% for both the empty liposomes and the extract-loaded ones. UV treatment did not affect weight loss in all the samples (*p* > 0.05). All the samples had a weight loss of about 0.5–2.2% until the beginning of the decomposition process. The reason for this is most likely the presence of volatile components in the samples that evaporated due to heating.

Based on the dTG curves, it can be concluded that the thermal decomposition of all the samples was a complex process. The decomposition of the pure rosehip fruit extract and extract-loaded liposomes took place in three steps (three peaks on the dTG curves), while for the empty liposomes, it took place in two steps (two peaks on the dTG curves). The first peak in the rosehip-fruit-extract-loaded liposome samples is due to the presence of extract, because this peak does not appear in the empty liposomes. In the case of biological materials, the first step of decomposition is the most important, because it is then that the degradation of biologically active components occurs. In the remaining steps, the thermal degradation of the substances created by the polymerization of the decomposition products created in the previous step takes place. T_on_ and T_p1_ values of the rosehip-fruit-extract-loaded liposomes were higher than those of the pure extract at about 70–73 °C and 86–92 °C, respectively, indicating that the encapsulation process significantly increased the thermal stability of the rosehip extract.

### 2.6. Polyphenol Release from Rosa canina Fruit Extract and Extract-Loaded Liposomes

**Figure 4:**
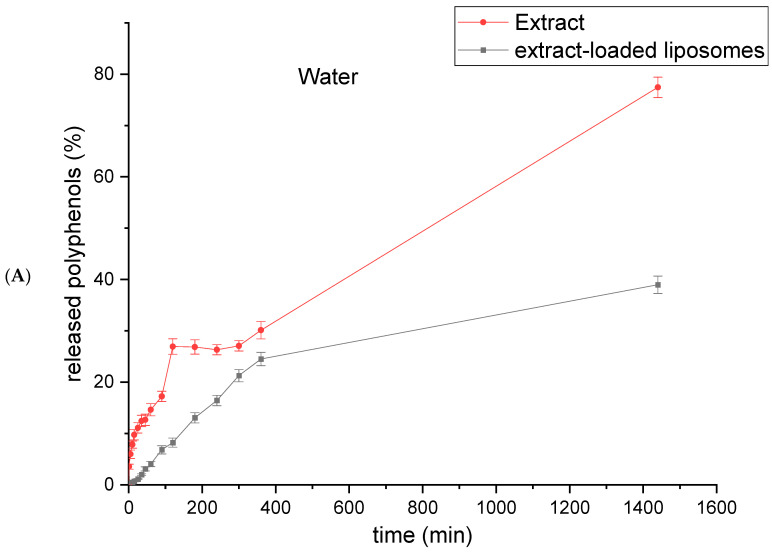

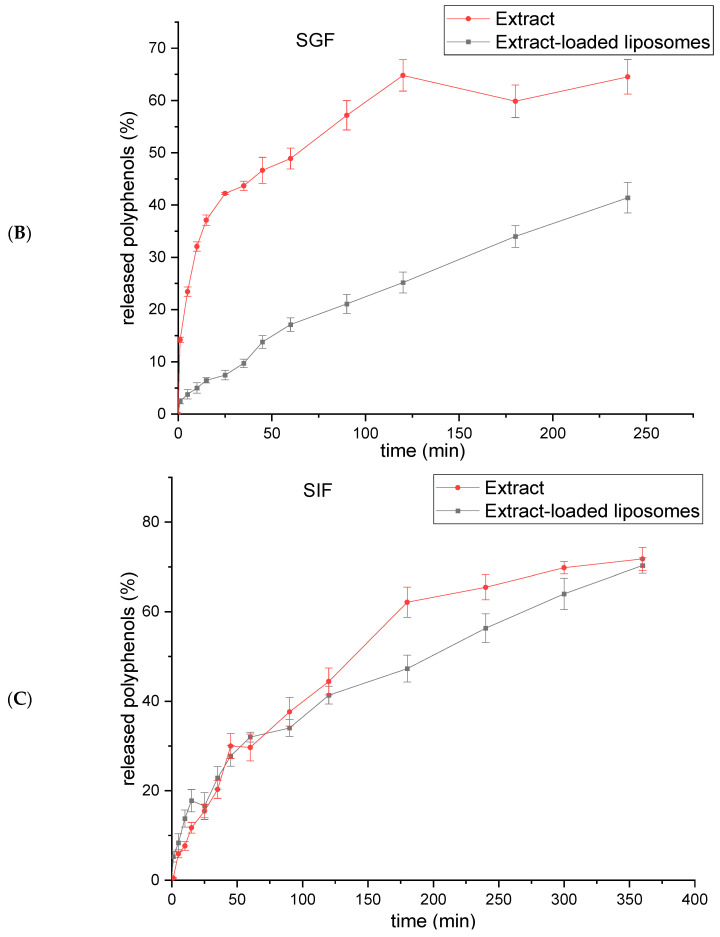
Kinetics of polyphenol release from the *Rosa canina* fruit ethanol extract and extract-loaded liposomes observed using Franz diffusion cells in (**A**) water at 25 °C, (**B**) simulated gastric fluid (SGF, pH 1.2) at 37 °C, and (**C**) simulated intestinal fluid (SIF, pH 6.8) at 37 °C.

In order to quantify the mass transfer resistance of the liposomal membrane, a polyphenol release study was carried out using a Franz diffusion cell in water at 25 °C and in SGF (pH 1.2) and SIF (pH 6.8) at 37 °C. The release curves of extract-loaded liposomes are compared with the curves of pure *R. canina* fruit extract diffusion (the same extract concentration as used for the liposome preparation) and presented in Figure 4.

As presented in Figure 4A, diffusion of polyphenols from the rosehip fruit extract occurred very quickly in water, and the quantity of polyphenols in the receptor compartment reached a plateau after 120 min. The release of polyphenols from the rosehip-fruit-extract-loaded liposomes in water was slower than expected. The steady state in water was not reached even after 360 min (Figure 4A). According to the presented results, liposomes can retain rosehip fruit polyphenols and, thus, be used for their prolonged release, which is significant for real applications.

The presence of pancreatin and bile salts (in SIF) enhanced the *R. canina* fruit polyphenol permeability from extract-loaded liposomes through a hydrophilic acetate cellulose membrane in the Franz diffusion cell; thus, the percentage of released polyphenols was statistically significantly higher (70.3 ± 1.7% after 6 h; Figure 4C) than the release in water after 24 h and in SGF after 4 h, which was 38.9 ± 1.6%, and 41.4 ± 2.9%, respectively (Figure 4A,B). In comparison with the liposomal samples, polyphenols from the pure rosehip fruit extract were released in higher amounts in water after 24 h (77.4 ± 2.0%) and in SGF after 4 h (64.5 ± 3.3%) (Figure 4A and 4B, respectively), whereas the percentage of polyphenols in the receptor compartment with SIF was the same as in the case of the liposomal sample (71.8 ± 2.6%) (Figure 4C). He et al. reported that the lipid bilayer is destroyed by pancreatic fluid, which contains lipolytic enzymes, such as lipases, phospholipase A_2_, and cholesterol esterase, while phospholipids are disrupted by bile salts [51]. Hence, the higher release of polyphenols in SIF was related to substantial enzymatic hydrolysis and bile-salt-induced degradation. The obtained results are in agreement with several publications demonstrating that liposomal membranes are structurally stable at lower pH levels, protecting hydrophobic and hydrophilic compounds from digestive degradation (limited release in gastric fluid), whereas the release in intestinal fluid is significant [52,53,54]. Nevertheless, Hu et al. showed that at an acidic pH, liposomes can maintain their integrity and pepsin cannot diffuse through the liposomal bilayer, but small molecules can be released due to liposomal membrane distortion at extremely low pH values [20].

Data obtained in the release studies were analyzed to determine the diffusion coefficients and diffusion resistances derived from liposomal membranes. The diffusion of polyphenols from liposomes to the receptor fluid through the membrane can be approximated using Fick’s second law, shown in Equation (1):

(1) $$\ln\;\left(\frac{{C_{d}^{0}-C}_{r}^{0}}{C_{d}{-C}_{r}}\right)=D\;\beta t,$$

where *C_d_* and *C_r_* are the concentrations of polyphenols detected in the donor and receptor compartments at time *t*; *C_d_*^0^ and *C_r_*^0^ are the concentrations of polyphenols at the start; and *D* is the diffusion coefficient. The geometrical constant *β* value, typical for the Franz cell geometry, was 2.49 × 10^4^ m^−2^ [55].

The diffusion coefficients of polyphenols from liposome dispersion were calculated from the slope of the linear part of a curve defined by plotting $\ln\;\left(\frac{{C_{d}^{0}-C}_{r}^{0\;}}{C_{d}{-C}_{r}}\right)$ vs. time *t* (Figure S2).

The overall diffusion resistance, *R* was calculated using Equation (2):

(2) $$R=\frac{\delta }{D},$$

where *δ* is the membrane thickness.

Diffusion resistance represents the cumulative resistance of a semipermeable acetate cellulose membrane and the resistance of a liposomal bilayer. The contribution of the resistance, which is generated by the synthetic membrane, was determined from the diffusion of polyphenols from the pure rosehip fruit extract. Then, the liposome resistance was determined by subtracting the synthetic membrane resistance from the overall diffusion resistance. The diffusion coefficients and diffusion resistance of the rosehip fruit extract and extract-loaded liposomes are presented in Table 4.

As can be seen in Table 4, the diffusion resistance was the highest for rosehip-fruit-extract-loaded liposomes in water, followed by extract-loaded liposomes in SGF and extract-loaded liposomes in SIF, whereas the lowest diffusion resistance was noted for the rosehip fruit extract in water. The presented results are acceptable from the point of view of the potential oral use of *R. canina*-fruit-extract-loaded liposomes because polyphenol release is not desirable in water (in the product) or in gastric fluid (in the stomach) but in the intestine, where the absorption of the largest quantity of polyphenols occurs. The obtained values of diffusion resistance were one, two, or even three orders of magnitude (10^6^ or 10^7^) higher compared with the resistance of resveratrol diffusion (10^5^) from phospholipid liposomes containing cholesterol [56] and of gentisic acid diffusion (10^4^) from phospholipid liposomes containing β-sitosterol [57]. This was expected, because liposomes containing sterols possess higher fluidity than the sterol-free liposomes in our case [58]. Additionally, *R. canina* fruit extract contains bigger molecules than resveratrol and gentisic acid; thus, these molecules are less penetrative.

## 3. Materials and Methods

### 3.1. Plant Material and Reagents

Dried rosehips were obtained from the Institute for Medicinal Plants Research “Dr. Josif Pančić”, Pančevo, Serbia. Identification of the plant was carried out by Dr. Slavoljub Tasić, Head of the Quality Control Sector at the Institute for Medicinal Plants Research “Dr. Josif Pančić”, and the plant was deposited in the Herbarium of the Faculty of Agriculture, Belgrade–Zemun, Serbia (voucher number: UBFA1353A-J). The following reagents were used: ethanol (Fisher Scientific, Loughborough, Leicestershire, United Kingdom), Phospholipon 90 G (unsaturated diacyl-phosphatidylcholine) (Lipoid GmbH, Ludwigshafen, Germany), hydrochloric acid, sodium chloride, potassium phosphate, sodium hydroxide, pepsin from porcine gastric mucosa (a peptidase), pancreatin from porcine pancreas (a mixture of several digestive enzymes: amylase, lipase, and protease) and bile salts (bile acid sodium salt, cholic acid–deoxycholic acid sodium salt mixture) were from Sigma-Aldrich, St. Louis, MO, USA.

### 3.2. Preparation of Rosa Canina Fruit Extract and Determination of the Extraction Yield

The harvested rosehip fruits were washed using water and kept in shadow to dry. The *R. canina* fruit ethanol extract was obtained using percolation at 25 °C, with the ground and sieved dried rosehip shells and 70% ethanol mixed in a 1:2 ratio; the pH value of the extract was 3.92. The rosehip fruit extract was stored at 4 °C for further analysis.

The extraction yield of the *R. canina* extract was calculated as follows:

(3) $$\mathrm{extraction}\;\mathrm{yield}\;\left(\%\right)=100-\frac{\left(a-b\right)*100}{m}$$

where *a* represents the weight (g) of the vessel containing the sample before drying, *b* represents the weight (g) of the vessel containing the sample after drying in Memmert 30–1060 (Memmert GmbH, Schwabach, Germany) at 105 °C to constant mass (approximately 2 h), and *m* represents the weight (g) of the sample. The extraction yield of the rosehip fruit ethanol extract was 32.8 ± 0.2%. The total polyphenol content of the rosehip fruit extract was previously published [59] and amounted to 9.64 mg gallic acid equivalent/mL of the liquid extract. In addition, the total flavonoid and protein contents in the rosehip fruit extract have also been published, amounting to 1.65 mg catechin equivalent/mL of the liquid extract and 0.827 mg albumin equivalent/mL of the liquid extract, respectively [60].

### 3.3. Preparation of Liposomal Vesicles and Lyophilization

Plain and *R. canina*-fruit-extract-loaded liposomes were prepared using a simple proliposome method described by Isailović et al. [56]. The method is based on the initial preparation of a proliposome mixture containing phospholipids, ethanol, and water, which is converted into liposomal spheres by a simple dilution step. Namely, 1 g of a phospholipid mixture (fatty flakes of phosphatidylcholine from soybean; Phospholipon) and 4 mL of 70% ethanol rosehip fruit extract (or 70% ethanol for plain liposomes) were measured in a beaker and stirred at 60 °C for 20 min on the laboratory magnetic stirrer with a PT 1000 temperature sensor (IKA RCT basic, IKA, Staufen, Germany) using a stir bar at a speed of 500 rpm. Due to the presence of ethanol (in the rosehip fruit extract) and high temperature, the phospholipids were dissolved, resulting in a molten mixture, while the heated beaker with the mixture was uncovered to allow the evaporation of ethanol. After ethanol evaporation and cooling, a proliposome mixture was obtained, and 20 mL of ultrapure water was then added in small portions in order to produce liposomal vesicles, *i.e.*, the proliposome mixture was converted into a liposomal dispersion due to the slow addition of water. Subsequently, the emulsion was stirred in a covered beaker at 800 rpm for 2 h (25 °C). Empty liposomes were prepared as a control; instead of the rosehip fruit extract, an extraction medium, i.e., 70% ethanol, was used for the dissolution of the phospholipids, after which the ethanol was evaporated using high temperature in an uncovered beaker for 20 min. After cooling, hydration was performed to formulate liposomal spheres containing only phospholipids without the extract. The liposomes were stored at 4 °C for further analyses.

With the aim of separating non-encapsulated fractions of polyphenols from the rosehip-fruit-extract-loaded liposomal population (for FTIR and HPLC analyses), the samples were centrifuged at 17,500 rpm and 4 °C for 45 min in a Thermo Scientific Sorval WX Ultra series ultracentrifuge (Thermo Scientific, Waltham, MA, USA).

For the FTIR and thermogravimetric analyses, lyophilized samples were prepared. To produce an extract sample, the ethanol was first removed at 50 mbar and 40 °C for 30 min using a Heizbad Hei-VAP rotary evaporator (Heidolph, Heidelberg, Germany). Then, all the samples were frozen in a LAB11/EL19LT freezer (Elcold, Hobro, Denmark) at −80 °C for 1 h and freeze-dried in a Beta 2–8 LD plus lyophilizer (Christ, Osterode am Harz, Germany) at −75 °C and 0.011 mbar for 24 h.

### 3.4. Storage-Stability Study

The size, PDI, zeta potential, mobility, and conductivity of plain and rosehip-fruit-extract-loaded liposomes were determined by dynamic light scattering (DLS) in a Zetasizer Nano Series, Nano ZS instrument (Malvern Instruments Ltd., Malvern, UK) and Malvern Dispersion Technology Software (DTS), version 7.03. Each sample was diluted 500-fold and measured three times at room temperature over 60 days of storage at 4 °C.

### 3.5. UV-Stability Study

One part of the prepared rosehip fruit extract was exposed to UV irradiation with the aim of investigating the influence of UV irradiation on the concentration of extract polyphenols, but this UV-irradiated part of the extract was not encapsulated in the liposomes. After the liposomal preparation, where the non-treated extract was encapsulated, the obtained liposomes (empty liposomes or extract-loaded liposomes) were separated into two parts. One part was exposed to UV irradiation, while the other part was non-treated to examine the influence of UV irradiation on the liposome characteristics. In a UV-stability study, 3 mL of *R. canina* fruit extract and plain and extract-loaded liposomes were exposed to UV irradiation (253.7 nm) in a thin layer for 20 min [61]; subsequently, DLS, density, surface tension, viscosity, TGA, HPLC, and FTIR analyses were performed.

### 3.6. Density, Surface Tension, and Viscosity Analyses

The density and surface tension of rosehip fruit extract and unloaded and extract-loaded liposomes (before and after UV irradiation) were measured using silicon crystal (as the immersion body) and Wilhelmy plates, respectively, in a Force Tensiometer K20 instrument (Kruss, Germany). Each sample was examined three times at room temperature.

The viscosity of non-treated and UV-irradiated unloaded and rosehip-fruit-extract-loaded liposomes, as well as of pure rosehip fruit extract, was determined using Rotavisc *lo-vi* device equipment with a VOL-C-RTD chamber, VOLS-1 adapter, and spindle (IKA, Staufen, Germany). Each sample (6.7 mL) was examined three times at room temperature.

### 3.7. Fourier-Transform Infrared (FTIR) Analysis

FTIR spectra of the initial components, pure Phospholipon, and pure rosehip fruit extract, as well as empty liposomes and extract-loaded liposomes (non-treated and UV-irradiated), were recorded in the transmission mode between 400 and 4000 cm^−1^ using the Nicolet iS10 spectrometer (Thermo Scientific, Uppsala, Sweden).

### 3.8. HPLC Analysis

HPLC analysis was performed using an Agilent 1260 RR HPLC instrument (Agilent, Waldbronn, Germany) equipped with a diode-array detector working in the range of 190–550 nm. The samples were separated using a reverse-phase Zorbax SB-C18 (Agilent) analytical column (150 mm × 4.6 mm i.d.; 5 μm particle size). Mobile phase A consisted of a 1% *v*/*v* solution of orthophosphoric acid in water, and mobile phase B consisted of acetonitrile. The gradient elution was performed as follows: 0–2.6 min, 90–85% A; 2.6–8 min, 85% A; 8–10.8 min, 85–80% A; 10.8–18 min, 80% A; 18–23 min, 80–70% A; 23–25 min, 70–50% A; 25–27 min, 50–20% A; 27–29 min, 20–10% A; 29–34 min, 0% A. Detection wavelengths were set at 350 nm, and the flow rate was 1 mL/min. The injection volume was 8 μL, and the column temperature was maintained at 40 °C. HPLC methodology was used for the determination of flavonols (quercetin, rutin, isoquercetin, and hyperoside) and one phenolic acid (chlorogenic acid). Identification of the compounds was achieved by comparing their UV spectra and retention time with those of authentic standard compounds (rutin, quercitrin, hyperoside, isoquercitrin, and chlorogenic acid). The amounts of the compounds were calculated using calibration curves. The results are expressed as micrograms per milliliter of rosehip fruit extract or liposomes (μg/mL).

### 3.9. Thermogravimetric Analysis

A thermogravimetric analyzer—TGA (TA Instruments TGA Q500, New Castle, Delaware, USA)—was used for the thermal analysis of samples. Thermograms were analyzed by the TA Advantage Universal Analysis 2000 software. The samples (8.0 ± 0.5 mg) were heated from room temperature to 500 °C at a heating rate of 10 °C/min under a nitrogen flow of 60 mL/min.

### 3.10. In Vitro Release Study

The in vitro release study was performed using a Franz diffusion cell (donated by PermeGear, Inc., Hellertown, PA, USA) with two compartments separated by an acetate cellulose membrane [62]. The study was conducted for pure *R. canina* fruit extract and extract-loaded liposomes in water in simulated gastric fluid (SGF) and in simulated intestinal fluid (SIF). SGF contained hydrochloric acid, sodium chloride, and pepsin (the pH was adjusted to 1.2 using hydrochloric acid), whereas SIF contained potassium phosphate, sodium hydroxide, pancreatin, and bile salts (the pH was adjusted to 6.8 using sodium hydroxide). The sample was placed in the donor compartment, while the receptor compartment was filled with medium (water or simulated fluid) and constantly mixed at 360 rpm by magnetic stirring at 25 °C for water and 37 °C for simulated fluids using a water jacket and a peristaltic pump. The release of polyphenols was monitored for 24 h (water), 4 h (SGF), and 6 h (SIF); the samples were taken from the receptor compartment at certain time intervals. The concentration of phenolic compounds in the samples was determined spectrophotometrically at 320 nm [63]. Samples were taken every five minutes and, later, every 30 min; because of the large number of samples generated, the direct spectrophotometric method was chosen for the determination of released phenolic compounds instead of the more expensive HPLC method.

The values of all the absorbances were determined on the UV-1800 UV spectrophotometer (Shimadzu, Kyoto, Japan).

### 3.11. Statistical Analysis

The statistical analysis was performed using analysis of variance (one-way ANOVA) followed by Duncan’s post hoc test within the statistical software, STATISTICA 7.0. The differences were considered statistically significant at *p* < 0.05 (n = 3). For the TGA analysis, measurements were performed in triplicate, and the results were shown as the mean ± SD. The XLSTAT (version 2014.5.03, Addinsoft, New York, NY, USA) analysis and statistics add-in for MS Excel (Version 2408) was used to calculate significant differences between the means by analysis of variance (ANOVA) followed by Tukey’s HSD test (*p* < 0.05).

## 4. Conclusions

Collectively, the data given in this study provide indications about the possibility of using the proliposome method for the preparation of liposomes with *R. canina*-sensitive polyphenolic compounds. This method was found to be convenient for the protection of polyphenols from adverse environmental conditions such as UV light. FTIR analysis confirmed that the plant compounds were successfully loaded within the liposomes, while no chemical reaction between rosehip fruit extract and phospholipids was detected, and neither were there changes caused by UV irradiation. The content of chlorogenic acid, rutin, and quercetin derivates (hyperoside, isoquercetin, and quercitrin) was similar in non-treated and UV-irradiated liposomal systems, evidencing the high potential for liposomal encapsulation to protect sensitive bioactives in rosehip fruit extract from the UV degradation. The size of rosehip-fruit-extract-loaded liposomes increased on the seventh day of storage, while the zeta potential values slightly varied in the same period and further stabilized during the 60 days of monitoring. The PDI of the rosehip-fruit-extract-loaded liposomes did not change during the storage period. Additionally, there were no significant changes in the density, surface tension, and viscosity between non-treated and UV-irradiated liposomal systems. UV treatment also did not affect the thermal stability of the liposomes, while the UV-treated rosehip fruit extract was less thermally stable than its non-treated parallel. *In Vitro* release studies in water and simulated fluids showed that the presence of enzymes and bile salts enhanced the rosehip–polyphenol permeability from liposomes in SIF compared with the release in water after 24 h and in SGF after 4h. The results of our study yielded many new insights that may be of interest to food manufacturers who intend to utilize liposomes as carrier systems for sensitive plant-derivate compounds as an alternative to synthetic antioxidants and conservans.

## Figures and Tables

**Table 1:** Density (*ρ*), surface tension (*γ*), and viscosity (*η*) of pure *Rosa canina* fruit extract, empty liposomes, and extract-loaded liposomes before and after UV irradiation.

Scheme	*ρ* [g/mL]	*γ* [mN/m]	*η* [mPa·s]
extract	0.931 ± 0.001 ^c^*	25.4 ± 0.2 ^a^	4.21 ± 0.01 ^c^
extract_uv_	0.969 ± 0.001 ^c^	23.3 ± 0.4 ^c^	5.49 ± 0.07 ^b^
liposomes	0.998 ± 0.000 ^b^	24.1 ± 0.1 ^b^	3.13 ± 0.01 ^d^
liposomes_UV_	0.997 ± 0.002 ^b^	24.4 ± 0.2 ^b^	3.14 ± 0.02 ^d^
extract-loaded liposomes	1.010 ± 0.002 ^a^	18.7 ± 0.3 ^d^	6.75 ± 0.05 ^a^
extract-loaded liposomes_UV_	1.012 ± 0.002 ^a^	18.5 ± 0.1 ^d^	6.71 ± 0.02 ^a^

* Values with the same letter (a–d) in each row showed no statistically significant difference (*p* > 0.05; n = 3; analysis of variance, Duncan’s post hoc test; mean value ± standard deviation); extract_UV_, UV-irradiated extract; liposomes_UV_, UV-irradiated empty liposomes; extract-loaded liposomes_UV_, UV-irradiated liposomes with extract.

**Table 2:** The content of polyphenol compounds in *Rosa canina* fruit extract and extract-loaded liposomes (before and after UV irradiation) determined using the HPLC method.

Sample	Chlorogenic Acid	Rutin	Quercetin Derivatives *
	µg/mL		
Extract	67.63 ± 1.42	157.81 ± 1.78	247.71 ± 2.63
Extract_UV_	57.42 ± 1.01	56.47 ± 1.14	135.63 ± 1.74
Extract-loaded liposomes	5.33 ± 0.65	18.83 ± 1.34	46.53 ± 1.86
Extract-loaded liposomes_UV_	6.32 ± 0.72	17.65 ± 1.22	48.14 ± 1.29

* Quercetin derivatives included hyperoside, isoquercetin, and quercitrin; extract_UV_, UV-irradiated extract; extract-loaded liposomes_UV_, UV-irradiated liposomes with extract.

**Table 3:** Results of the thermogravimetric analysis of pure *Rosa canina* fruit extract, empty liposomes, extract-loaded liposomes, and their UV-treated counterparts.

	Extract	Extract_UV_	Liposomes	Liposomes_UV_	Extract-Loaded Liposomes	Extract-Loaded Liposomes_uv_
WL until TD	0.5 ± 0.1 ^b^	0.5 ± 0.2 ^b^	1.8 ± 0.4 ^a^	2.2 ± 0.5 ^a^	1.8 ± 0.4 ^a^	1.9 ± 0.6 ^a^
TD						
T_on_ (°C)	108.5 ± 1.5 ^c^	104.1 ± 1.9 ^d^	228.4 ± 0.5 ^a^	225.3 ± 1.7 ^a^	177.8 ± 0.6 ^b^	177.4 ± 1.1 ^b^
T_off_ (°C)	386.2 ± 1.1 ^a^	386.2 ± 1.8 ^a^	376.5 ± 0.6 ^bc^	372.4 ± 1.7 ^d^	377.6 ± 1.6 ^b^	373.7 ± 1.0 ^cd^
WL (%)	58.4 ± 0.6 ^b^	59.0 ± 1.3 ^b^	81.8 ± 0.9 ^a^	80.7 ± 0.9 ^a^	80.0 ± 0.7 ^a^	80.0 ± 1.3 ^a^
T_p1_ (°C)	125.9 ± 1.7 ^b^	121.8 ± 0.8 ^c^	-	-	212.0 ± 1.9 ^a^	214.1 ± 1.4 ^a^
T_p2_ (°C)	197.0 ± 1.0 ^c^	195.0 ± 0.5 ^c^	268.2 ± 1.2 ^a^	268.4 ± 1.4 ^a^	266.6 ± 1.0 ^ab^	265.5 ± 0.6 ^b^
T_p3_ (°C)	309.2 ± 0.9 ^c^	310.4 ± 1.5 ^c^	332.3 ± 1.1 ^a^	331.4 ± 1.6 ^a^	328.4 ± 1.8 ^ab^	326.5 ± 1.5 ^b^
Residue 500 °C	35.8 ± 0.9 ^a^	35.2 ± 0.9 ^a^	11.8 ± 0.9 ^b^	12.0 ± 0.6 ^b^	11.4 ± 0.7 ^b^	11.3 ± 1.0 ^b^

Values are presented as the mean ± standard deviation (n = 3), and different lowercase superscripts within the same row indicate a significant difference in the means according to Tukey’s honest significant difference (HSD) test (*p* < 0.05). WL, weight loss; TD, thermal decomposition; T_on_, onset temperature; T_off_, offset temperature; T_p_, peak temperature; extract_UV_, UV-irradiated extract; liposomes_UV_, UV-irradiated empty liposomes; extract-loaded liposomes_UV_, UV-irradiated liposomes with extract.

**Table 4:** Diffusion coefficients (*D*) and diffusion resistance (*R*) of the *Rosa canina* fruit extract and extract-loaded liposomes in water, simulated gastric fluid (SGF), and simulated intestinal fluid (SIF).

Conditions	Sample	*D* [m^2^/s]	*R* [s/m]
Water, 25 °C	Extract	2.07 × 10^−9^	1.47 × 10^6^
	Extract-loaded liposomes	3.35 × 10^−10^	1.90 × 10^7^
SGF, pH 1.2, 37 °C	Extract	2.54 × 10^−8^	1.20 × 10^5^
	Extract-loaded liposomes	1.67 × 10^−9^	3.80 × 10^6^
SIF, pH 6.8, 37 °C	Extract	7.50 × 10^−9^	4.07 × 10^5^
	Extract-loaded liposomes	4.88 × 10^−9^	1.30 × 10^6^

## Data Availability

The datasets generated during and/or analyzed during the current study are available from the corresponding author upon reasonable request.

## Supplementary Materials

The following supporting information can be downloaded at: https://www.mdpi.com/article/10.3390/plants13182608/s1, Figure S1: HPLC chromatogram of polyphenol compounds in *Rosa canina fruit extract* (a—non-treated and b—UV-irradiated) and extract-loaded liposomes (c—non-treated and d—UV-irradiated); detection wavelengths of 350 nm; numbers related to individual compound names—1, chlorogenic acid, 2, rutin, 3, hyperoside, 4, isoquercetin, and 5, quercitrin.; Figure S2: The dimensionless plot of *Rosa canina* fruit extract polyphenol concentration in water, simulated gastric fluid (SGF), and simulated intestinal fluid (SIF) vs. time for the release curves; e, extract; L, liposomes, SGF, simulated gastric fluid; SIF, simulated intestinal fluid; C_d_ and C_r_, concentrations of *R. canina* fruit polyphenols in donor and receptor compartments at time t, C_d_^0^, and C_r_^0^, concentrations of *R. canina* fruit polyphenols at the beginning of the analysis.
