# Supplementary material for: Design and Characterization of Liposomal-Based Carriers for the Encapsulation of Rosa canina Fruit Extract: In Vitro Gastrointestinal Release Behavior
Source: Plants (Basel). 2024 Sep 18;13(18):2608. doi: 10.3390/plants13182608 (PMC11435264; doi:10.3390/plants13182608)
Supplement: Supplementary file 1 [file plants-13-02608-s001.zip › plants-3159044-supplementary.pdf]

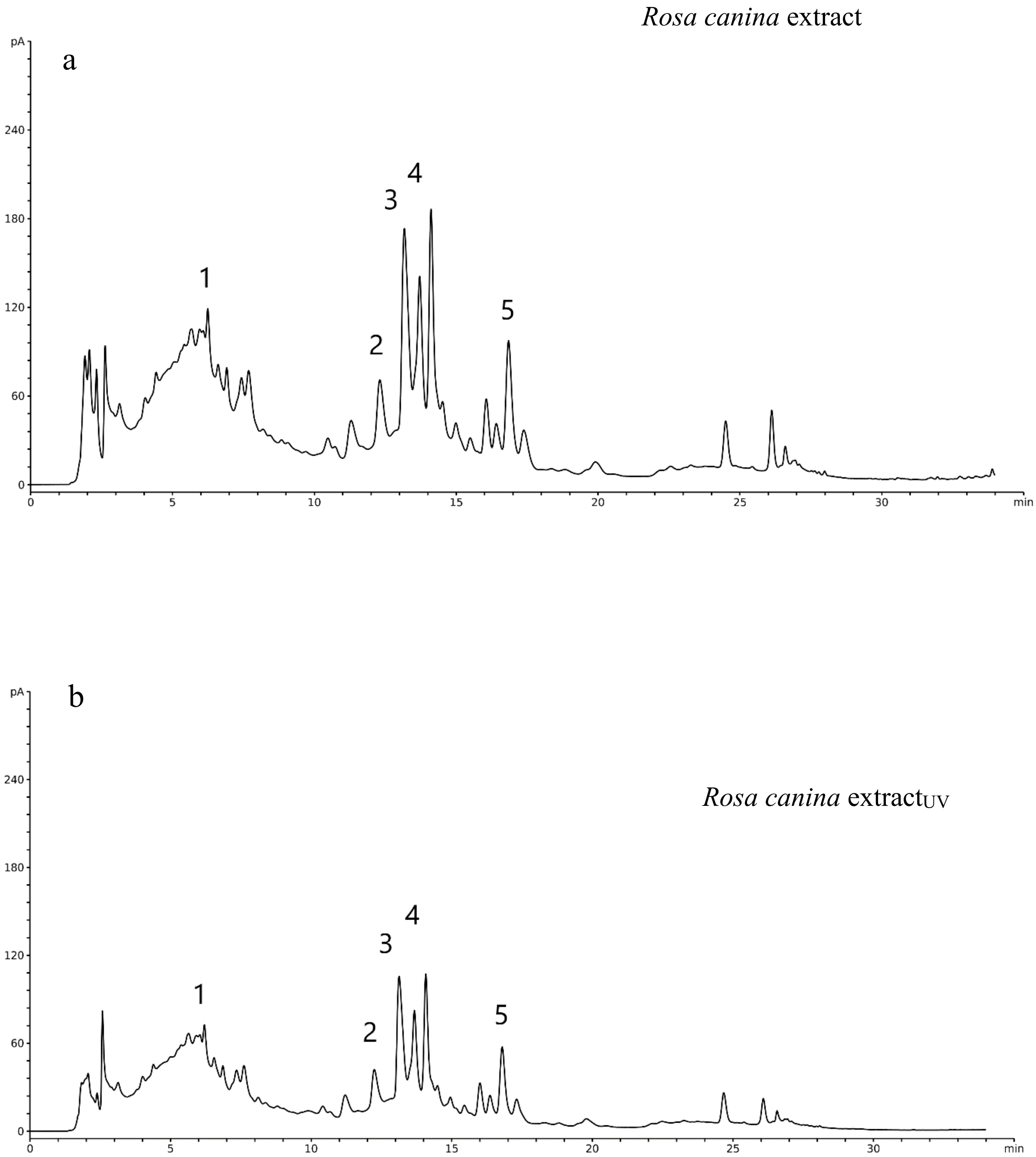

Rosa canina extract
a
pA
240
180
120
60
0
1
2
3
4
5
0
5
10
15
20
25
30
min
b
pA
240
180
120
60
0
Rosa canina extractUV
1
2
3
4
5
0
5
10
15
20
25
30
min

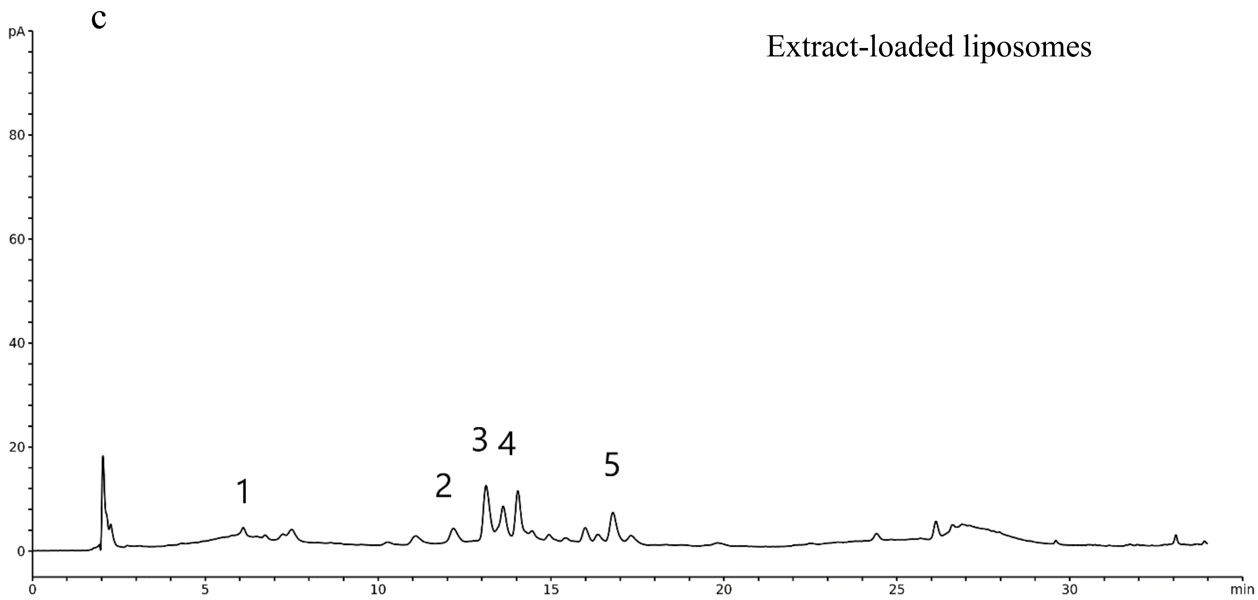

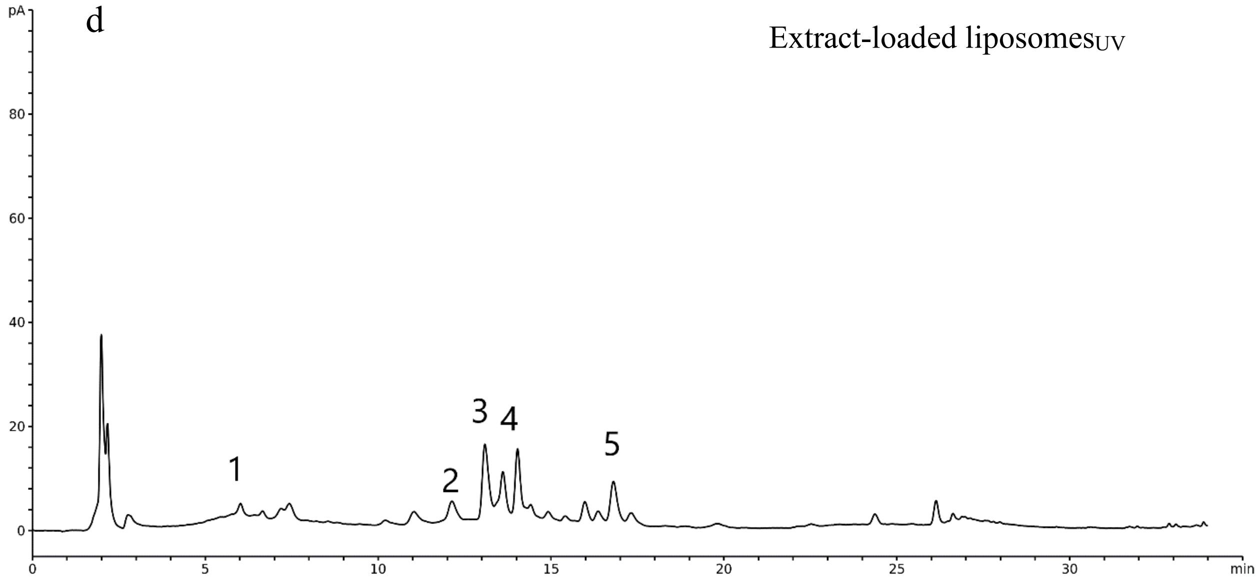

Supplement 1. HPLC chromatogram of polyphenol compounds in *Rosa canina* fruit extract (a - non-treated and b - UV-irradiated) and extract-loaded liposomes (c -non-treated and d - UV-

irradiated); detection wavelengths of 350 nm; numbers related to individual compound names - 1, chlorogenic acid, 2, rutin, 3, hyperoside, 4, isoquercetin, and 5, quercitrin.

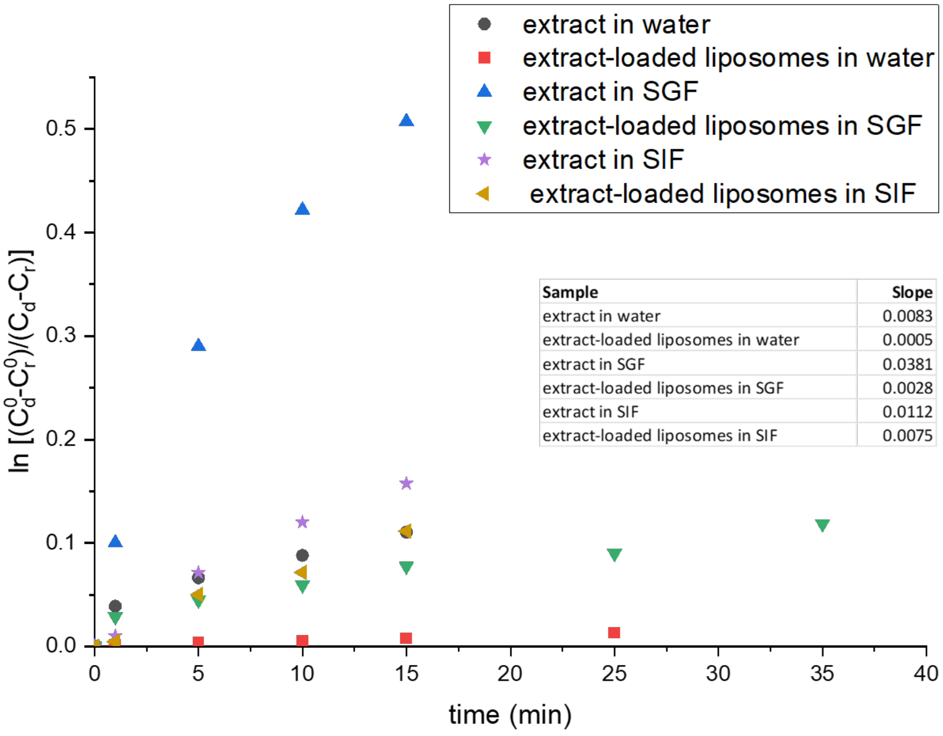

Supplement 2. The dimensionless plot of *Rosa canina* fruit extract polyphenol concentration in water, simulated gastric fluid (SGF), and simulated intestinal fluid (SIF) *vs.* time for the release curves; e, extract; L, liposomes; C_d and C_r, concentrations of *R. canina* fruit polyphenols in donor and receptor compartments at time *t*, $C_d^{0,}$ and C_r^{0}, concentrations of *R. canina* fruit polyphenols at the beginning of the analysis.
